# Level of implementation of WHO COVID-19 document on rights, roles and responsibilities of health care workers in a tertiary hospital in Southwest Nigeria

**DOI:** 10.11604/pamj.supp.2020.37.1.26078

**Published:** 2020-12-29

**Authors:** Oluseyi Ademola Adejumo, Oludamilola Adebola Adejumo, Oghenekaro Godwin Egbi, Olatunji Sunday Abolarin, Oladimeji Emmanuel Alli

**Affiliations:** 1 Department of Internal Medicine, University of Medical Sciences Teaching Hospital Complex, Ondo City, Ondo State, Nigeria,; 2 Department of Business Law, Faculty of Law, Obafemi Awolowo University, Ile-Ife, Osun State, Nigeria,; 3 Department of Internal Medicine, Niger Delta University, Bayelsa State, Nigeria

**Keywords:** Rights, roles, responsibilities, health care workers, World Health Organization, COVID-19

## Abstract

**Introduction:**

in order to curb the increasing spread of COVID-19 amongst health care workers (HCWs), the World Health Organization (WHO) released the COVID-19 Rights, Roles and Responsibilities of Health workers (COVID-19 RRRHW) policy document aimed at protecting HCWs amidst the on-going pandemic. This study determined the level of implementation of the document in a tertiary hospital in Southwest Nigeria.

**Methods:**

this was a cross-sectional descriptive study among HCWs in a tertiary hospital in South-western Nigeria that assessed the level of implementation of the WHO COVID-19 RRRHW document using a closed ended structured questionnaire.

**Results:**

five hundred and thirty-five HCWs participated in the study comprising 165(30.8%) males and 370(69.2%) females. Majority (87.3%) of the HCWs were 40 years and below. One hundred and sixty-three (30.5%) of the HCWs had been involved in the care of COVID-19 patients; less than 60% of the respondents were aware of the presence of an official platform for dissemination of information on suspected or confirmed cases of COVID-19 and 435(81.3%) were aware of hospital training on Infection Prevention and Control (IPC); 191(35.7%) have had uninterrupted supplies of personal protective equipment(PPE) and IPC materials; 211(39.4%) were aware of mental and counselling services in the hospital while only 106(19.8%) knew how to access these services; 289(54%) have attended IPC training and 307(57.4%) are able to don and doff PPE.

**Conclusion:**

this study showed inadequate implementation of the WHO COVID-19 RRRHW document. There is urgent need for all stakeholders to familiarize with the document in order to ensure adequate protection of HCWs and minimize their risk of contracting COVID-19.

## Introduction

The coronavirus pandemic has undoubtedly changed the course of events globally since its outbreak in December, 2019. The outbreak which started in Wuhan, China has gradually spread to 215 countries [1]. The World Health Organization (WHO) declared the coronavirus as a pandemic in March 2020 [2]. Over 22,208,491 cases and 781,536 deaths have been recorded globally as at 20^th^ August, 2020 [1]. The first case of the virus was recorded in Nigeria in February 2020 in an Italian citizen who came into the country on a flight from northern Italy [3]. Between February and August, a total of 49, 895 cases have been confirmed in 36 States of the federation and the Federal Capital Territory with 981 deaths recorded as at 18^th^ August, 2020 [4]. The increase in the number of cases has not been without consequences as facilities and personnel required for management of patients are getting strained. This pandemic has placed huge pressure on Nigeria´s already fragile healthcare system which has been further aggravated by the mass exodus of medical practitioners in recent times. As much as there is need to pay attention to health care delivery and access to care for coronavirus disease-19 (COVID-19) patients and suspected cases, in keeping with the United Nations Sustainable Development Goal of Universal Health Coverage (UHC) [5], it is equally crucial that the rights of health care workers (HCWs) at the forefront of managing the pandemic are equally adequately protected. The need to ensure protection of HCWs was equally also emphasized by the International Partnership for Universal Health Coverage 2030 (UHC2030) [6]. Relying on earlier studies, Shah *et al*. [7] reported a total number of 1,716 infected health personnel as of February 11, 2020 [8]. The WHO in early May 2020, reported infection of 945 HCWs in 28 counties in the African region with South Africa having the highest number of infected HCWs. Before mid-May 2020, Nigeria has become the most affected in the African region with 401 HCWs infected [9]. By 26^th^ May, 2020, Nigeria still retained the highest number with 606 HCWs having been infected with the coronavirus [9].

With the increasing number of infected HCWs, additional pressure is being put on the other HCWs in various hospitals to put in extra hours to cover the work of those who are ill or on isolation. It is thus imperative that adequate measures are put in place to protect them. To this end, the WHO recognizing the need to protect HCWs as frontline workers in combating the COVID-19 pandemic, released the COVID-19 outbreak: rights, roles and responsibilities of health workers, including key considerations for occupational safety and health document in March 2020 (COVID-19 RRRHW document) [10]. The COVID-19 RRRHW document contains both rights and corresponding responsibilities. It highlights the responsibilities of employers of HCWs; responsibilities of HCWs and the rights of HCWs. Whilst the responsibilities on the part of the employers and HCWs are not unusual, the rights of HCWs which can be reasonably implied from the employer´s responsibilities create a clear departure from the usual norm. The usual focus has always been on protection of patients who are considered vulnerable in the medical practitioner-patient relationship especially from the view point of medical ethics which Draper and Sorell accurately described as being 'one-sided' [11]. The increased vulnerability of HCWs to infection in this COVID-19 era clearly justifies the need to highlight, recognize and implement their rights. The following rights of HCWs can be clearly inferred from the COVID-19 RRRHW document: 1) Right to information, instruction and training on occupational safety and health (OSH) and infection prevention and control (IPC); 2) Right to adequate supply of IPC and personal protective equipment (PPE) and training on use; 3) Right to a blame-free environment which allows HCWs to freely report incidents relating to exposures, cases of violence and the right of victims of violence and exposures to needed support; 4) Right to report symptoms and to stay home when ill without being required to return to work where there is continuing or serious danger to life or health, until the employer has taken the necessary remedial action; 5) Right to appropriate working hours with breaks; 6) Right to abstain from work without undue consequences where there is reasonable justification to believe that the situation at work presents an imminent and serious danger to their life or health; 7) Right to compensation, rehabilitation and treatment if infected with COVID-19 following exposure in the workplace; and 8) Right to access mental health and counselling services.

Consistent with the above rights, the employers of HCWs have corresponding responsibilities to ensure that measures are put in place to prevent violation of these rights. The COVID-19 RRRHW document also imposes clear obligations on HCWs including responsibility to follow established OSH procedures; to avoid exposure of others to health and safety risks; to participate in employer-provided OSH trainings; to use provided protocols to assess, triage and to treat patients; to treat patients with respect, compassion and dignity; to maintain patient confidentiality; to swiftly follow established public health reporting procedures of suspected and confirmed cases; to provide or reinforce accurate IPC and public health information to patients and the community; to put on, use, take off and dispose of PPEs properly; to self-monitor for signs of illness and self-isolate or report illness to managers, if it occurs; to inform management if they are experiencing signs of undue stress or mental health challenges that require support interventions; and to report to their immediate supervisor any situation which they have reasonable justification to believe presents an imminent and serious danger to life or health [10]. Notwithstanding the release of the COVID-19 RRRHW document in March, 2020, the rate of infection of HCWs in Nigeria has continued to increase [9]. The success of the COVID-19 RRRHW document as a tool for protecting HCWs in the COVID-19 era is however, dependent on the extent to which HCWs and indeed all stakeholders are aware of, and committed to the implementation of this document. The aim of this study therefore, was to assess the practices and measures put in place to reduce the spread of COVID-19 in University of Medical Sciences Teaching Hospital Complex, (UNIMEDTHC) Ondo State, Southwest Nigeria during the pandemic and determine the level of implementation of WHO-COVID-19 RRRHW policy document. To the best of our knowledge, we are not aware of any similar study conducted in Nigeria. We believe the study will be relevant in identifying gaps in successful implementation of the COVID-19 RRRHW document and recommending measures to be taken in creating awareness and sensitization toward guaranteeing improved protection of HCWs in Nigeria in this COVID-19 era.

## Methods

This was a cross-sectional descriptive study that assessed the level of implementation of the COVID-19 RRRHW document at UNIMEDTHC, Ondo, South-western Nigeria which is a State owned tertiary hospital. Suspected patients with COVID-19 who present at this hospital are managed until the diagnosis is confirmed. The confirmed patients with COVID-19 are subsequently transferred to the State government designated treatment centre known as Infectious Disease Hospital (IDH) once there is available bed space. The study was carried out in the month of July 2020. A total of 600 questionnaires were distributed among HCWs in the hospital. The respondents included both clinical and non-clinical staff of the hospital. Administrative staff of the hospital were excluded.

**Data collection:** the level of implementation of the COVID-19 RRRHW document was assessed through the use of close-ended structured questionnaire that comprised 45 questions. The questionnaire had sections A, B, C and D. Section A consisted of questions on socio-demographic information and medical history of the HCWs while Section B, C and D consisted of questions on the roles and responsibilities of the hospital management to HCWs; the roles and responsibilities of the HCWs; and rights of the HCWs based on the COVID-19 RRRHW document.

**Ethical consideration:** ethical clearance was obtained from the Ethical and Research Committee of University of Medical Sciences, Ondo State. Informed consent was obtained from each participant. All questionnaires were coded (without names), and confidentiality of responses was ensured throughout the study.

**Data analysis:** data generated were analyzed using the Statistical Package for the Social Sciences for Window version 20.0 (SPSS Inc., Chicago, IL, USA). Results were presented in tabular form and discrete variables were presented as frequency and percentages.

## Results

A total of 570 questionnaires were returned with a response rate of 95%. At the end of the study, only 535 questionnaires were analyzed because 35 questionnaires were excluded due to incomplete filling. There were 535 HCWs in this study comprising 165(30.8%) males and 370(69.2%) females. Majority (87.3%) of the HCWs were 40 years and below. Among the respondents, 180(33.6%) were nurses, 124(23.2%) were doctors and 97(18.1%) were health assistants. Amongst the respondents, 135(25.2%) worked in the Department of Internal Medicine, 86(16.1%) in the Department of Surgery and 61(11.4%) in the Emergency Department. Four hundred and twelve (77%) of the respondents had tertiary or postgraduate qualification. Three hundred and eighty two (71.4%) have been working in the hospital for 5 years and below. Working experience was ≤10years in 438(81.9%) of the respondents (Table 1). The identified co-morbidities among the respondents were hypertension (5.2%), asthma (4.1%), and diabetes mellitus (0.6%). One hundred and sixty-three (30.5%) of the HCWs had been involved in the care of COVID-19 patients; 97(18.1%) had gone into self-isolation following contact with COVID-19 patient(s); 46.2% (247) have had reason to work for extra hours since onset of the pandemic; 98(18.3%) reported work situation as posing threat to their health while 217(40.6%) had been assaulted by patients or their caregivers in the past (Table 2).

**Table 1:** socio-demographic characteristics of participants (N =535)

Socio-demographic characteristics	N (%)
**Gender**	
Males	165(30.8)
Females	370(69.2)
**Age group**	
≤40 years	467(87.3)
>40years	68(12.7)
**Occupation**	
Nurse	180(33.6)
Doctor	124(23.2)
Health assistant	97(18.1)
Cleaners	44(8.2)
Others	90(16.9)
**Departments**	
Internal Medicine	135(25.2)
Surgery	86(16.1)
Emergency	61(11.4)
Obstetrics and Gynecology	39(7.3)
Paediatrics	32(6.0)
Dentistry	24(4.5)
Laboratory	19(3.6)
Pharmacy	15(2.8)
Physiotherapy	14(2.6)
Others	110(20.6)
**Religion**	
Christianity	493(92.1)
Islam	37(6.9)
Traditional	1(0.2)
Others	3(0.6)
Not stated	1(0.2)
**Level of Education**	
None	3(0.6)
Primary	20(3.7)
Secondary	100(18.7)
Tertiary	298(55.7)
Post-graduate	114(21.3)
**Work experience Post qualification**	
≤10years	438(81.9)
>10years	84(15.7)
Not stated	13(2.4)
**Average working hours per week**	
≤60	354(66.2)
>60	163(30.5)
Not stated	18(3.4)
**Duration of employment in the hospital**	
≤5years	382(71.4)
>5years	142(26.5)
Not stated	11(2.1)

**Table 2:** risk assessment of study participants

QUESTIONS	YES n(%)	NO n(%)	NOT SURE n(%)
Are you hypertensive?	28(5.2)	492(92.0)	15(2.8)
Are you diabetic?	3(0.6)	522(97.6)	10(1.9)
Are you asthmatic?	22(4.1)	505(94.4)	8(1.5)
Does your department have confirmed case(s) of COVID-19?	146(27.3)	355(66.4)	34(6.4)
Have you had to care for a suspected case of COVID-19?	163(30.5)	335(62.6)	37(6.9)
Have you gone on self-isolation after exposure to a patient with COVID-19?	97(18.1)	438(81.9)	0(0)
Has any staff tested positive to corona virus after exposure to a confirmed case(s)?	27(5.0)	386(72.1)	122(22.8)
Have you had to work extra hours during this COVID-19 pandemic?	247(46.2)	282(52.7)	6(1.1)
Have you reported any work situation as posing danger/threat to health?	98(18.3)	426(79.6)	0(0)
Have you ever been assaulted by patient/caregiver in the course of carrying out your duty?	217(40.6)	314(58.7)	4(0.7)

Less than 60% of the respondents were aware of presence of platform for dissemination of information on suspected or confirmed cases of COVID-19 and information update on COVID-19; 435(81.3%) were aware of hospital training on IPC; and about one-third were aware of the non-existence of a dedicated COVID-19 response team. Two hundred and eleven (39.4%) were aware of mental health and counseling services in the hospital, but only 106(19.8%) knew how to access these services (Table 3). Sixty-nine (12.6%) of the respondents claimed that surgical face masks were always available for use in the hospital. Four hundred and twenty-four (79.3%) of the respondents claimed that the hospital had uninterrupted supply of running water while 406(75.9%) of the respondents reported constant availability of soap in the hospital. One hundred and ninety-one (35.7%) have had uninterrupted supplies of needed PPE and IPCs materials while 286(53.5%) have had to buy PPEs with their personal money to attend to patients (Table 4). Two hundred and eighty-nine (54%) of the respondents have attended IPC training while 174 (32.5%) have attended OSH training. Three hundred and seven (57.4%) are able to don and doff PPE; 355(66.4%) have counselled patients, caregivers and the community on COVID-19 in the past. On the other hand, 158(29.5%) respondents could not adequately attend to patients because of lack of PPE (Table 5).

**Table 3:** frequency of answers to questions on responsibilities of the hospital during COVID-19 pandemic

QUESTION	YES n(%)	NO n(%)	I DON'T KNOW n(%)
Provision of a platform for disseminating information on suspected/confirmed COVID-19 cases	253(47.3)	190(35.5)	92(17.2)
Provision of updated information on COVID-19	296(55.3)	207(38.7)	32(6.0)
Organization of IPC training	435(81.3)	58(10.8)	42(7.9)
Organization of training on donning and disposal of PPE	294(55.0)	219(40.9)	21(3.9)
Existence of a dedicated COVID-19 response team	197(36.8)	179(33.5)	159(29.7)
Presence of a protocol for reporting exposure to bodily fluids of patients/cases of violence	172(32.1)	184(34.4)	179(33.5)
Provision of compensation/rehabilitative services	20(3.7)	324(60.6)	191(35.7)
Availability of a mental and counseling department	211(39.4)	135(25.2)	189(35.3)
Provision of access to mental health services	106(19.8)	330(61.7)	99(18.5)
Presence of a protocol for reporting suspected COVID-19 cases	319(59.6)	109(20.4)	107(20.0)

**Table 4:** availability of PPE and IPC materials in the hospital

QUESTION	Always available n(%)	Sometimes available n(%)	Never available n(%)	Don't know n(%)
Availability of cloth facemask	247(46.2)	250(46.7)	25(4.7)	13(2.4)
Availability of surgical facemask	69(12.9)	290(54.2)	159(29.7)	17(3.2)
Availability of respirator	21(3.9)	120(22.4)	340(63.6)	54(10.1)
Availability of face shield	230(43.0)	221(41.3)	70(13.1)	14(2.6)
Availability of gowns	81(15.1)	169(31.6)	223(41.7)	62(11.6)
Availability of hand sanitizer	237(44.3)	262(49.0)	31(5.8)	5(0.9)
Availability of hand-washing soap	406(75.9)	110(20.6)	13(2.4)	6(1.1)
Availability of running water	424(79.3)	95(17.8)	9(1.7)	7(1.3)
Availability of cleaning supplies	327(61.1)	173(32.3)	14(2.6)	21(3.9)
	**Yes (%)**	**No (%)**	**Not stated (%)**	
Uninterrupted PPE and IPC supplies	191(35.7)	329(61.5)	15(2.8)	
Individual purchase of PPE/IPC materials by HCWs for patients' care	286(53.5)	232(43.4)	17(3.2)	

**Table 5:** frequency of answers to questions on responsibilities of the HCWs during COVID-19 pandemic

QUESTIONS	YES n(%)	NO n(%)	I DON'T KNOW
Did you attend any training on infection prevention and control?	289(54.0)	191(35.7)	55(10.3)
Did you participate in the training on occupational safety and health?	174(32.5)	353(66.0)	8(1.5)
Do you know how to don/doff personal protective equipment (PPE)?	307(57.4)	224(41.9)	4(0.7)
Have you neglected any patient because of unavailability of personal protective equipment (PPE)?	158(29.5)	363(67.9)	14(2.6)
Have you educated any patient/caregiver about COVID-19?	355(66.4)	168(31.4)	12(2.2)

## Discussion

This study showed inadequate implementation of the WHO COVID-19 RRRHW document at UNIMEDTHC, Ondo, Nigeria. There were more female participants in this study. This may be explained by the fact that 60% of the participants were nurses, health assistants and cleaners who are more likely to be females. This also corroborates WHO report on gender and health workforce statistics showing a higher proportion of female workers in the health sector [12]. In our study, nurses and doctors constituted a higher proportion of the HCWs. Majority of the HCWs were 40 years and below which may be due to the fact that the teaching hospital where the study was conducted is a relatively young health institution. Also, majority of the respondents have tertiary or postgraduate qualification which is expected in tertiary health institution offering specialist health care services; undergraduate and postgraduate trainings for medicine and other related courses like physiotherapy, medical laboratory science and nursing.

**Medical co-morbidities and risk assessment among HCWs:** the prevalence rates of identified co-morbidities such as hypertension, diabetes mellitus and asthma among the HCWs in this study were low. The relatively low prevalence of hypertension and diabetes mellitus may be due to the fact that majority of the study population were young adults. Established risk factors for severe form of COVID-19 such as hypertension, diabetes mellitus and older age were not prevalent among our respondents [13-16]. The implication is that the likelihood of having severe form of COVID-19 and associated mortality among this workforce is low.

**Presence of COVID-19 response team and infection prevention control committee (IPCC):** there is no COVID-19 response team in UNIMEDTHC as recommended by WHO COVID-19 RRHW document; there is however an IPCC. Despite the absence of a response team, about one-third of the respondents erroneously believed there was a COVID-19 response team at the hospital. The absence of a response team may be due to the fact that the hospital is not a treatment centre for COVID-19 patients. Patients with confirmed diagnosis of COVID-19 are usually transferred to the State government owned IDH. However, there have been instances where confirmed patients could not be immediately transferred to IDH due to non-availability of bed spaces. Such patients were managed in the hospital by the IPCC and other HCWs until they are transferred to IDH. As at the time of this study, about a third of the HCWs had been directly or indirectly involved in the care of COVID-19 patients while about 18% had gone into self-isolation following contact with COVID-19 patient(s). It is strongly believed that the constitution of a COVID-19 response team in the hospital as recommended by WHO will significantly reduce risk of exposure of HCWs to suspected or confirmed COVID-19 patients.

**Provision of PPE and IPC supplies:** about a third of the HCWs had uninterrupted supplies of PPE and IPC supplies while about half have had to personally purchase PPE to protect themselves while attending to patients at the time of this study. Similarly, over 90% of the HCWs observed that running water supply, soap and hand sanitizer were sometimes or always available. Although fairly commendable, however there is a need for the hospital management to work towards uninterrupted supply of these IPC supplies at all service points in order to limit spread of infection within the hospital. Previous reports have alluded to the fact that there was a global challenge with uninterrupted supplies of PPE in both developing and developed countries [17-19]. Inadequate supplies of PPEs were also reported among HCWs in United States, United Kingdom, Africa, Pakistan and India [17-21]. Factors responsible for this global challenge included limited financial resources to procure, shutting down of manufacturing companies during the pandemic, panic purchase of large quantity of PPE by people in anticipation of future hike in price and out of stock and hoarding of PPEs by business owners in order to maximize profit. There have been insinuations that this shortage of PPE contributed to high rate of COVID-19 infection and mortality among HCWs in different countries. The State government has however taken commendable steps to increase availability of PPE to hospitals by engaging in local production of protective gowns and mask, mass purchase of respirators which is currently being made available to HCWs within the State.

**IPC training:** the hospital management organized training on IPC for different cadres of HCWs at different times. Some key participants such as heads of unit were supposed to organize step-down training in their different units. Majority of the HCWs were aware of IPC training that was conducted by the hospital which showed that the training programmes were adequately publicized. However, only about half of the HCWs claimed to have attended the IPC training. This suggests that there is a need to monitor and evaluate activities of those saddled with the responsibility of conducting step down training for members of their respective units. Also, the IPC training should be more regular in order to maximize attendance and the benefits of the program.

**Dissemination of information:** less than half of the respondents were aware of a platform for disseminating information on suspected or confirmed cases of COVID-19; about 60% of the respondents were aware of a protocol for reporting suspected COVID-19 cases and 55.3% were aware of a platform where updates are provided for HCWs. There is need for the hospital to make information disseminating platforms and protocols accessible to all HCWs in order to respect their right of access to information and to equip them with information and materials that will be useful in educating the community. However, it must be emphasized that while information is being disseminated, confidentiality of information about patients and HCWs with COVID-19 must be respected.

**Workplace violence:** a total of 40.6% of the HCWs claimed they had been abused by patients or their relatives in the course of discharging their professional duties in the past. This is lower than 58.2% reported among Ethiopian HCWs by Yenealem *et al*. [22] and 56.4% reported among Chinese HCWs by Tian *et al*. [23]. Workplace violence among HCWs have received considerable attention of the WHO because of the negative impact it has on their fundamental human rights; psychological and physical well-being; productivity and job satisfaction [24]. This could further compound the burden already imposed on these HCWs in the present COVID-19 pandemic. The hospital management needs to put in place adequate measures that will protect the rights of HCWs and protect them from work place violence by possibly adopting and implementing the WHO framework guidelines for addressing workplace violence in the health sector [24].

**Compensation and rehabilitation:** the WHO RRRHW document recommended that there should be provision of compensation for HCWs in this pandemic. The compensation may be for those who had to put in extra hours beyond what is expected of them, those who are frontline HCWs in the management of COVID-19, those who become infected in the line of duty, and those who eventually succumb to COVID-19. This is meant to encourage them and allay the fears of what the future holds in case they succumb to COVID-19 or suffer any permanent injury in the line of duty. Following the onset of COVID-19, 46.2% of our study population had worked for extra hours; 18.1% had gone on self-isolation following contact with confirmed cases of COVID-19 and 30.5% had been involved in the care of suspected COVID-19 patients. This showed the level of risk HCWs are exposed to during the pandemic and the need for adequate compensation. However, the process of compensation and rehabilitation has not been set in motion in the hospital possibly because it goes beyond what could be handled at the level of hospital management without government intervention. This issue requires holistic approach that would involve formulation and implementation of policies; legislation of laws; and executive approval by the government. Presently, the frontline workers in the state owned IDH do not have concrete insurance plans to guarantee their safety in the course of discharging their responsibilities. In Nigeria, HCWs have always lamented the lack of government´s commitment to their welfare. This also came to the fore in the middle of the COVID-19 pandemic when it became public knowledge that the hazard allowance paid to Nigerian HCWs was less than 150 USD per annum. After several consultations, the federal government agreed to pay HCWs 50% of their basic salary as COVID-19 allowance for three months. Despite this agreement, the government still failed to pay the allowance until the Association of Resident Doctors embarked on industrial action which almost paralyzed the health sector and with fatal consequences amidst the COVID-19 pandemic [25]. Even till date, several State government HCWs have not received their allowances and others are planning to embark on industrial action. This further compounds the problem of managing the COVID-19 pandemic in Nigeria where increasing number of new cases and deaths are recorded daily.

**Mental health of HCWs:** in this study, less than half of the respondents were aware of the existence of mental health and counselling unit in the hospital, while majority of the respondents claimed they do not have access to mental health and counselling department. The hospital has not put a formal structure in place to provide psychological support for HCWs. HCWs especially those taking care of patients with COVID-19 are at increased risk of developing psychological disorders such as stress, insomnia, anxiety and depression [26-29]. Factors responsible for increased psychological burden among HCWs include absence of definitive cure for COVID-19; high infectivity of the virus; limited PPE; fear of getting infected by the virus and subsequently exposing friends and family members to the disease; and increased working hours. Also, HCWs are faced with discrimination and stigmatization in the society where the people see them as a potential source of the infection [30,31]. In addition, the peculiarity of long waiting time between sample collections and collection of results may also contribute to the psychological burden of HCWs. It takes an average of one week for COVID-19 polymerase chain reaction test result to come out due to limited COVID-19 diagnostic facilities in Southwest Nigeria. This waiting time is characterized by apprehension, agitation, anxiety and palpable fear of the unknown. Therefore, it is highly imperative that the psychological needs of HCWs should be prioritized and met. The WHO advocates provision of mental support and counseling to HCWs. Xiao *et al*. [27] reported that psychological support given to frontline HCWs during the COVID-19 pandemic improved their quality of sleep, self-efficacy and reduced their level of stress and anxiety. In line with the WHO COVID-19 RRRHW document, the hospital needs to ensure that the HCWs are aware of the availability of mental health services while also reinforcing the counselling unit. Accessibility to these services should be improved upon so that HCWs can have their psychological needs met during the pandemic. This will enable them to be more effective in the discharge of their duties.

**Responsibilities of HCWs:** about half of the HCWs had attended training on IPC while less than two-third claimed they could properly don and doff PPE. This proportion is relatively low bearing in mind that majority of HCWs in this study were clinical staff. It is expected that even HCWs who do not work directly with patients should also be trained on how to don and doff PPE, because the exigencies of the pandemic may require them to work as volunteers in the clinical units. Also, only a third of the respondents had undergone training on OSH. There is need to improve on this aspect of training, because it is essential for HCWs to know how to stay safe at their work place while caring for patients, especially those with COVID-19. About two-third of respondents had been involved in the education of patients, relatives and the larger community on COVID-19. However, because the opinions of HCWs are highly respected and valued in the communities, there is a need to maximize their positive influence in community. They can assist in emphasizing the importance of regular hand washing, social distancing, cough etiquette and also help to debunk the myths surrounding COVID-19. The limitation of this study was that it was a quantitative study which relied on recall of information by respondents; therefore, there may be some form of bias in their responses.

## Conclusion

The rate of infection of HCWs in Nigeria has continued to increase since the first COVID-19 case was discovered in Nigeria in February, 2020.The release of the COVID-19 RRRHW document by the WHO was to guarantee increased protection of the rights and welfare of HCWs amidst the pandemic. However, this study revealed low level of implementation of the document with need for increased attention by the government and all stakeholders in ensuring the protection of the rights and welfare of HCWs. Issues of compensation and rehabilitation of HCWs; protection of HCWs from violence; access to counselling services to ensure the mental well-being of HCWs; access to information, training, adequate PPEs and IPC for their protection are areas requiring urgent attention. Implementation of the document will guarantee improved protection of rights of HCWs with a consequential decrease in rate of infection among them.

**Recommendations:** the need for increased effort to ensure that delay between sample collection and release of COVID-19 test results of HCWs is addressed cannot be over-emphasized. This will not only prevent prolonged anxieties, but will also ensure that HCWs who are positive are immediately isolated without exposing their patients and other HCWs to risk of infection. This study recommends the creation of a proper and formal structure to prevent violence against HCWs in the discharge of their duties and to provide psychological support and counselling for HCWs to enable them cope with increased stress in the discharge of their duties during the pandemic. Also, IPC trainings should be more regularly conducted with mandatory step-down trainings by heads of respective units. A dedicated COVID-19 response team that will be responsible for the care of COVID patients in the hospital should also be created to limit spread of infection among HCWs. Lastly, there is need for increased commitment to the welfare of HCWs on the part of the government. Government should not allow matters to degenerate to the level of strike actions before key demands relating to the welfare and well-being of HCWs are met.

### What is known about this topic

- The COVID-19 is an on-going global pandemic that has devastating effect on the health care personnel and infrastructure of affected countries;
- There is increasing rate of COVID-19 infection among HCWs globally;
- The WHO released the COVID-19 RRRHW document in March, 2020 which is aimed at protecting HCWs and curbing the spread of COVID-19 amongst HCWs in the course of discharging their professional duties.

### What this study adds

- There is low level of implementation of the COVID-19 RRRHW policy document; hence concerted efforts should be geared towards its publicity among stakeholders;
- Issues of HCWs' compensation and rehabilitation; protection from violence; access to mental health and counseling services; access to information, training, adequate PPEs and IPC materials for their protection require urgent attention.

## References

[1] World Health Organization Coronavirus disease (COVID-19) weekly epidemiological update and weekly operational update.

[2] New York Times Coronavirus has become a pandemic, WHO says 11^th^ March 2020.

[3] Ebenso B, Otu A (2020). Can Nigeria contain the COVID-19 outbreak using lessons from recent epidemics?. Lancet Glob Health.

[4] Nigeria Centre for Disease Control COVID-19 situation report -situation report 172 Tuesday 18 ^th^ August 2020.

[5] United Nations “Sustainable Development Goals Knowledge Platform”.

[6] Kickbusch I, Gitahi G COVID-19 (coronavirus): Universal health coverage in times of crisis.

[7] Shah K, Kamrai D, Mekala H, Mann B, Desai K, Patel RS (2020). Focus on mental health during the coronavirus (COVID-19) pandemic: applying learnings from the past outbreaks. Cureus.

[8] World Health Organization COVID-19 WHO African Region: External situation report 10.

[9] World Health Organization COVID-19 WHO African Region: External situation report 11.

[10] World Health Organization Coronavirus disease (COVID-19) outbreak: rights, roles and responsibilities of health workers, including key considerations for occupational safety and health document.

[11] Draper H, Sorell T (2002). Patients´ responsibilities in medical ethics. Bioethics.

[12] World Health Organization Sources and classification of health workforce statistics.

[13] Rastad H, Karim H, Ejtahed HS, Tajbakhsh R, Noorisepehr M, Babaei M (2020). Risk and predictors of in-hospital mortality from COVID-19 in patients with diabetes and cardiovascular disease. Diabetol Metab Syndr.

[14] Albitar O, Ballouze R, Ooi JP, Ghadzi SM (2020). Risk Factors for Mortality among COVID-19 Patients. Diabetes Res Clin Pract.

[15] Huang S, Wang J, Liu F, Liu J, Cao G, Yang C (2020). COVID-19 patients with hypertension have more severe disease: a multicenter retrospective observational study. Hypertension Research.

[16] Li X, Xu S, Yu M, Wang K, Tao Y, Zhou Y (2020). Risk factors for severity and mortality in adult COVID-19 in patients in Wuhan. J Allergy Clin Immunol.

[17] Gondi S, Beckman AL, Deveau N, Raja AS, Ranney ML, Popkin R (2020). Personal protective equipment needs in the USA during the COVID-19 pandemic. Lancet.

[18] Osseni IA (2020). COVID-19 pandemic in sub-Saharan Africa: preparedness, response, and hidden potentials. Trop Med Health.

[19] Ahmed J, Malik F, Arif TB, Majid Z, Chaudhary MA, Ahmad J (2020). Availability of Personal Protective Equipment (PPE) Among US and Pakistani Doctors in COVID-19 Pandemic. Cureus.

[20] Aljazeera News UK admits PPE shortage amid coronavirus criticism.

[21] The BMJ Opinion COVID-19: Indian healthcare workers need adequate PPE.

[22] Yenealem DG, Woldegebriel MK, Olana AT, Mekonnen TH (2019). Violence at work: determinants & prevalence among health care workers, northwest Ethiopia: an institutional based cross sectional study. Ann Occup Environ Med.

[23] Tian Y, Yue Y, Wang J, Luo T, Li Y, Zhou J (2020). Workplace violence against hospital healthcare workers in China: a national WeChat-based survey. BMC Public Health.

[24] World Health Organization Violence and injury prevention: violence against health workers.

[25] The Guardian Nigerian News Tension as resident doctors begin strike amid COVID-19.

[26] Spoorthy MS, Pratapa SK, Mahant S (2020). Mental health problems faced by healthcare workers due to the COVID-19 pandemic-a review. Asian J Psychiatr.

[27] Xiao H, Zhang Y, Kong D, Li S, Yang N (2020). The effects of social support on sleep quality of medical staff treating patients with coronavirus disease 2019 (COVID-19) in January and February 2020 in China. Med Sci Monit.

[28] Cai H, Tu B, Ma J, Chen L, Fu L Jiang, Zhuang Q (2020). Psychological impact and coping strategies of frontline medical staff in Hunan between January and March 2020 during the outbreak of coronavirus disease 2019 (COVID19) in Hubei, China. Med Sci Monit.

[29] Kang L, Li Y, Hu S, Chen M, Yang C, Yang BX (2020). The mental health of medical workers in Wuhan, China dealing with the 2019 novel coronavirus. Lancet Psychiatry.

[30] Bagcchi S (2020). Stigma during the COVID-19 pandemic. Lancet Infect Dis.

[31] Singh R, Subedi M (2020). COVID-19 and stigma: social discrimination towards frontline healthcare providers and COVID-19 recovered patients in Nepal. Asian J Psychiatr.

